# Genotype Diversity of *Mycobacterium bovis* and Pathology of Bovine Tuberculosis in Selected Emerging Dairy Regions of Ethiopia

**DOI:** 10.3389/fvets.2020.553940

**Published:** 2020-09-30

**Authors:** Getnet Abie Mekonnen, Adane Mihret, Mekdes Tamiru, Elena Hailu, Abebe Olani, Abde Aliy, Melaku Sombo, Matios Lakew, Balako Gumi, Gobena Ameni, James L. N. Wood, Stefan Berg

**Affiliations:** ^1^National Animal Health Diagnostic and Investigation Center, Sebeta, Ethiopia; ^2^Animal Health and Zoonotic Research Unit, Aklilu Lemma Institute of Pathobiology, Addis Ababa University, Addis Ababa, Ethiopia; ^3^Bacterial and Viral Diseases Research Directorate, Armauer Hansen Research Institute, Addis Ababa, Ethiopia; ^4^Department of Veterinary Medicine, College of Food and Agriculture, United Arab Emirates University, Al Ain, United Arab Emirates; ^5^Disease Dynamics Unit, Department of Veterinary Medicine, University of Cambridge, Cambridge, United Kingdom; ^6^Animal and Plant Health Agency, Weybridge, United Kingdom

**Keywords:** *Mycobacterium bovis*, spoligotype, cattle, Ethiopia, lesion distribution

## Abstract

Bovine tuberculosis (bTB) is endemic in Ethiopia with higher prevalence in cattle, particularly in the central parts. Spread of *Mycobacterium bovis (M. bovis)* to wider regions is inevitable in uncontrolled conditions. This study was conducted to explore the pathology, characterize *M. bovis* strains, and describe genotypic diversity to demonstrate possible epidemiological links in emerging dairy areas of Ethiopia, namely, Mekelle and Gondar. Twenty-seven bTB positive cattle identified by the Single Intradermal Comparative Cervical Tuberculin (SICCT) test were subjected to post-mortem inspection to determine lesion distribution and pathological score. Samples from tissues with visible tuberculous or suspected non-visible lesions were processed and cultured following a standard protocol. Isolates identified as *M. bovis* by Region of Difference (RD)-based Polymerase Chain Reaction (PCR) were also spoligotyped to determine their spoligotype patterns. Post-mortem inspection of visceral organs indicated bTB suggestive lesions in 41% of the animals, with 25% being in the lungs. Lymph nodes from 77% of the animals had lesions. Fifty-five isolates identified from 24 of the slaughtered animals were confirmed as *M. bovis*. No other mycobacterial species were isolated. Spoligotyping classified strains from 21 of these animals into seven spoligotype patterns: SB0133, SB0134, SB1176, SB2233, SB2290, SB2467, and SB2520. More than one spoligotype were identified from five of these animals, and none of the last four spoligotypes had been reported in Ethiopia before. SB0134 was the most predominant type (47%) followed by SB0133 (25.5%). SB0133, SB2290, SB2467, and SB1176 are spoligotypes lacking spacers 3–7, characteristics of *M. bovis* strains of the African 2 (Af2) clonal complex, while SB0134, SB2233, and SB2520 do not belong to any of the established clonal complexes and likely to have a different evolutionary history. Despite a small sample size, the present study showed strain diversity with multiple genotypes identified in a single herd and even within a single animal, and the genotypes showed no sign of geographical localization, which could be a consequence of significant movement of bTB diseased cattle around the country, spreading the disease. Therefore, any future control programme of bTB in Ethiopia needs to address the risks of cattle movement.

## Introduction

Bovine tuberculosis (bTB), caused primarily by *Mycobacterium bovis* (*M. bovis*), is a chronic disease affecting mainly cattle but also a wide range of hosts including other domesticated animals, certain wildlife species, and human beings (1, 2). The geographic spread of *M. bovis* corresponds primarily to the distribution of livestock throughout the world (3). In developed countries, eradication programmes have significantly reduced the prevalence of bTB although the spillover from wildlife reservoirs makes complete eradication difficult (4, 5). However, control is more challenging in low income countries, where the disease continues to cause significant losses in the cattle farming sector due to lower productivity (6), with implications for public health due to potential zoonotic transmission (7).

In Ethiopia, the overall skin test prevalence of bTB in cattle is estimated at 5.8% with a wide range of variation between regions, production systems, and breed compositions (8). For example, the intensive dairy sector in the central part of Ethiopia has animal level prevalence recorded between 22 and 47% (9–13). The dairy breed composition in this region is largely Holstein-Frisian and their crosses with Zebus managed in intensive or semi-intensive systems. These breeds have been reported to be more susceptible to *M. bovis* infection (12). In the peripheral regions of Ethiopia, the prevalence in the dairy sector has been increasing progressively as a consequence of dairy expansion to fulfill the need for dairy products arose from the growing human population. Studies in these areas showed prevalence between 1.4 and 13.6% (14–18).

The demand for improved dairy cattle stock for expanding dairy systems in peripheral regions has been met mainly by the acquisition of cross bred dairy cattle from the central areas of the country. Such centrifugal trading, from the central part of the country where the disease burden is higher, could contribute to disease spread. The association of bTB with the cattle movement network has been described recently in (19). However, genotyping of *M. bovis*, which could demonstrate epidemiological links, has been insufficient. This study was undertaken to explore the extent of tuberculous lesions in Single Intradermal Comparative Cervical Tuberculin (SICCT) reactor cattle in different regions of Ethiopia and to characterize the diversity of *M. bovis* strains in those animals using spoligotyping.

## Materials and Methods

### Study Sites

The study was conducted in two cities, namely, Gondar and Mekelle, supposed to represent the emerging dairy areas of the northern and north-western parts of Ethiopia, aimed to explore the bTB situation in these regions. These urban centers held more than 700 dairy herds comprising of 7,400 dairy cattle, from which 59 and 61 herds comprising of 976 and 818 cattle from Gondar and Mekelle were considered for the study, respectively. In addition to the dairy herds in these areas, a dairy herd owned by the Agricultural, Technical and Vocational Education Training College (ATVET) at Alage, 215 km south of Addis Ababa, was also included in the study as it was one of the biggest herds (*n* = 284) that was distributing dairy animals to the southern and east central parts of the country, with potential risk of disease transmission. The investigated cattle herds were managed in intensive and semi-intensive systems, the majority of which were established within the last 10 years. The husbandry and farm settings differed somewhat from one site to the other depending on the level of awareness and experience on dairy farming.

### Ante- and Post-mortem Examination

bTB infected cattle for post-mortem examination were identified based on the 2016/17 survey aimed to estimate the prevalence and associated risk factors (14) using tuberculin antigens with standard interpretation—positive if the difference of skin thickness increase at the bovine site (PPD-B) was greater by 4 mm than the increase at avian site (PPD-A) of the SICCT test (20). Animals were selected based on the willingness of farmers to offer reactor cattle for a fair compensation or to consciously cull reactors without compensation to avoid further transmission within their herds. Accordingly, 27 out of 2,078 (22%) animals were selected. However, the authors would like to admit the possibility of bias introduction to some degree due to non-randomized selection and small sample size. Ante-mortem inspection of these animals was conducted before they were slaughtered for post-mortem inspection by an experienced pathologist, and relevant data were recorded. Age of the animals was determined based on dentition pattern; body condition score (BCS) was determined and categorized into three scales (poor, medium, and good) by modifying the five scales described by Kellogg (21) to better reflect the assessment in field conditions.

In post-mortem, each lobe of the lungs, liver, spleen, intestines, and kidneys were inspected externally and then sliced at a slice-thickness of 10–20 mm as appropriate to facilitate detection of any tubercle lesions. Lymph nodes of the head region—mandibular, parotid, retropharyngeal; thoracic region—pre-scapular, tracheobronchial, mediastinal; abdominal region—portal, mesenteric; and inguinal and thigh region—pre-femoral, supra-mammary were sliced into 2–5 mm sections and inspected for presence of typical tubercle lesions. Gross lesions were scored using a semi-quantitative approach as described by Ameni et al. (22) and Vordermeier et al. (23). Briefly, rated as “Score 3” when the lesions were multifocal and coalescing; “Score 2” when the lesions were multifocal but not coalescing; “Score 1” when the lesion was in one focus (just starting the tubercle lesion); and “Score 0” when there was no visible lesion. Lesion scoring was performed by the same operator for all animals to ensure scoring consistency. Summation of the pathological scores was considered to determine the total pathological score at the animal level. Furthermore, the type, scale, and size of the lesions were recorded.

### Tissue Sample Collection

Tissue samples from organs and tissues suspected of tubercle lesions identified during the post-mortem inspection were collected from all slaughtered cattle. Collected tissue samples were placed in 50 ml sterile universal tubes (with sterile PBS buffer) and kept on ice until and during transport to the laboratory where samples were stored at −20°C for 1 week and then at −80°C if processing was performed later than 1 week after collection.

### Sample Processing for Culture

Samples were processed according to a standard protocol described in Roberts et al. (24) but with modification on the neutralization step by adding PBS instead of HCl. Tissues were dissected, manually crashed and homogenized using a pestle and mortar, followed by decontamination in an equal volume of 4% NaOH for 15 min with frequent shaking. The sample mix was then filled to 50 ml with PBS buffer and centrifuged at 1,750 × g. The sediments were neutralized by refilling to 50 ml with PBS and concentrated by centrifugation at 1,750 × g for 15 min. The pellets were re-suspended with 1 ml PBS and then inoculated with 3–5 drops in triplicate of Löwenstein-Jensen (LJ) medium slants, i.e., two slants supplemented with pyruvate and the other with glycerol. The slants were incubated at 37°C for 8 weeks; slants were examined on a weekly basis to check for mycobacterial growth.

Cultures were considered negative if no visible growth was detected after 8 weeks of incubation. Microscopic examination of cultures using the Ziehl–Neelsen staining method was performed to identify acid-fast bacilli. Heat-killed cells of each isolate were prepared by mixing colonies in 500 μl distilled H_2_O followed by incubation at 80°C for 1 h. Acid-fast positive cultures were preserved as stocks for future use in Dubos Tween-albumin broth and stored at −80°C.

### Genomic DNA Extraction and RD4 Deletion Typing

Extraction of genomic DNA was made from heat-killed cultures using a Qiagen DNA extraction kit according to the manufacturer's instruction (25). RD4 deletion typing, a chromosomal deletion characteristic of *M. bovis*, was made using conventional PCR (26). PCR amplification was performed in a total volume of 27 μL consisting of 12.5 μL HotStarTaq Master Mix (Qiagen, United Kingdom), 3.5 μL Qiagen water, 2 μL of each oligonucleotide primer (10 μM), and 5 μL DNA template. The RD4 oligonucleotide primers used included: RD4-FlankFW 5′- CTC GTC GAA GGC CAC TAA AG - 3′, RD4-FlankRev 5′- AAG GCG AAC AGA TTC AGC AT - 3′, and RD4-InternalFW 5′- ACA CGC TGG CGA AGT ATA GC - 3′ (27). The PCR condition involved amplification in a FlexCycler^2^ (Analytikjena, Germany), using an initial denaturation step at 96°C for 15 min, followed by 35 cycles of denaturation at 96°C for 30 s, annealing at 55°C for 1 min, and elongation at 72°C for 30 s. Cycling was completed by a final elongation step at 72°C for 10 min. The reaction product was resolved by electrophoresis on a 1.5% agarose gel (1.5 g agarose in 100 mL TAE buffer) containing SYBR safe stain (Invitrogen) for examination using a gel documentation system (BioRad Laboratories, USA). Identified strains of *M. bovis* and *Mycobacterium tuberculosis* were used as positive controls (PCR products of 446 bp (RD4 deleted) and 335 bp (RD4 present), respectively (27) and Qiagen water was employed as a negative control for deletion typing.

### Mycobacterial Strain Differentiation

Mycobacterial isolates identified as *M. bovis* by RD4 deletion typing were further confirmed as *M bovis* and genotyped by spoligotyping as described by Kamerbeek et al. (28). PCR amplification of spacers were accomplished using two primers, namely, DRa (5′-GGT TTT GGG TCT GAC GAC-3′, biotinylated) and DRb (5′-CCG AGA GGG GAC GGA AAC-3′) in a thermal cycler (Applied Biosystem) using 35 cycles of denaturation at 96°C for 1 min, annealing at 55°C for 1 min and elongation at 72°C for 30 s. The primers were designed to anneal to all repeat sequences and thereby enabling amplification of all spacers that occur in the DR region of a specific strain. The amplified spacers were then hybridized with the known oligonucleotide spacers covalently bound to a non-commercial “biodyne C membrane,” produced by APHA (UK) according to Kamerbeek et al. (28). Hybridization was done by incubating the membrane for 1 hr at 60°C. Hybridized DNA was detected by incubating the membrane in enhanced chemiluminescence (ECL) detection fluid (GE Healthcare) for 2 min and visualized by exposing a light sensitive ECL film for 20 min. The membrane was then analyzed by recording the presence or absence of signals at the sites of the individual probes (29).

Genomic DNA of known *M. bovis* and H37Rv were used as positive controls for *M. bovis* and *M. tuberculosis*, respectively. Identified spoligotype patterns were compared at a global database of *M. bovis* genotypes (http://www.mbovis.org) (30). The discriminatory power of spoligotyping, i.e., the average probability that this assay assigns a different type for two unrelated strains randomly sampled among the available *M. bovis* isolates, was assessed by using Hunter-Gaston Diversity Index (HGDI) (31) using *in silico* website of the University of the Basque Country: http://insilico.ehu.es/mini_tools/discriminatory_power/index.php.

## Results

### Description of Selected Cattle

**Figure 1:**
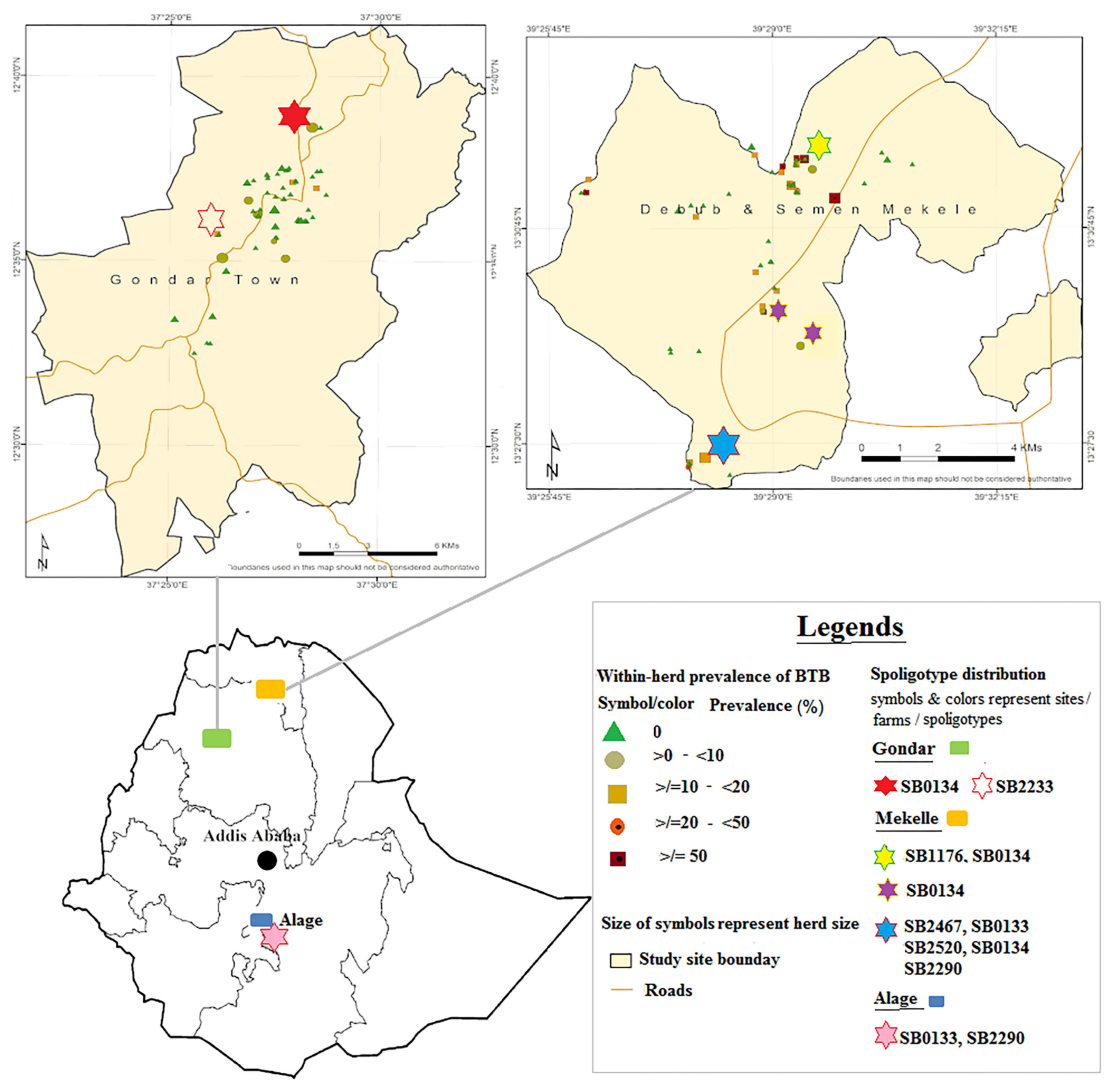
Distribution of *Mycobacterium bovis* spoligotypes by study sites and farms. Source: http://maplibrary.org/library/stacks/Africa/Ethiopia/index.htm.

The total number of SICCT reactor cattle was 122 out of 2,078 tested cattle kept in 33 herds, from which 27 reactors (two male and 25 female) were recruited from 10 herds for post-mortem inspections. Seventeen animals were from four herds in Mekelle, and five animals were from five herds in Gondar. The remaining five reactor cattle were donated by Alage ATVET (Figure 1). The number of animals tested in this herd was 284, of which eight were reactors for the SICCT test. Animals sourced from Gondar and Mekelle were crosses of Holstein Frisian and Zebu breeds, while cattle from Alage were all Boran cattle (Zebu breed). In ante-mortem, three animals were highly emaciated and showed chronic cough, one animal had a swollen parotid lymph node on its right side, while the remaining animals showed no clinical signs of being diseased.

### Lesion Description and Pathological Scoring

**Figure 2:**
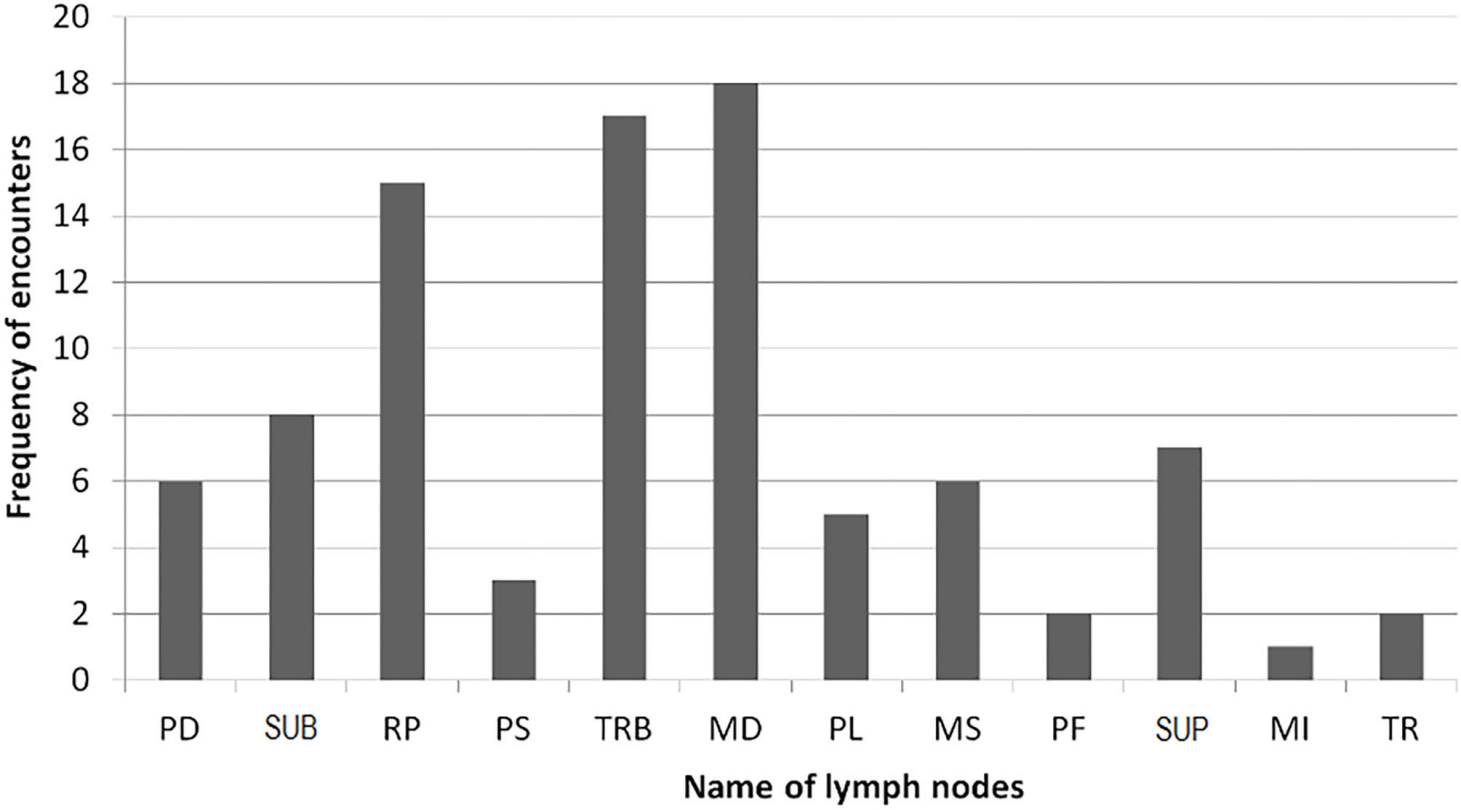
Frequency of lesion encounter in lymph nodes (see footnote under Table 1 for the abbreviations).

Post-mortem inspection showed visible tuberculous lesions in 21 out of 27 slaughtered cattle; the remaining six cattle showed no visible lesions in any of their organs or lymph nodes. All 21 animals identified with visible lesion(s) were suggestive of having tuberculosis in the lymph nodes. A total of 90 lesions were observed in the examined lymph nodes, with the most frequently affected being in mediastinal (MD), tracheobronchial (TRB), and retropharyngeal (RP) lymph nodes (Figure 2). Comparison of the total pathological scores for the examined lymph nodes also showed that higher scores were observed for these three lymph nodes, while the least was for medial iliac and prefemoral lymph nodes (Table 1).

**Table 1:** Lesion descriptions and scoring for selected lymph nodes of 21 animals.

**Description of lesions**		**Name of lymph node**^****a****^ **(*****n*** **=** **21 animals)**											
		**PD**	**SM**	**RP**	**PS**	**TRB**	**MD**	**PL**	**MS**	**PF**	**SM**	**MI**	**TR**
Type of lesion	Mucoid	2	1	6	2	7	3	3	–	2	1	–	2
	Purulent	2	1	1	–	1	3		1	–	–	–	1
	Caseous	1	3	4	1	7	8	1	3	–	–	1	1
	Calcified	1	2	2	–	2	3	1	2	–	1	–	–
Scale of lesion	Single	2	1	2	–	5	3	1	1	1	1	–	–
	Multiple	2	3	6	3	7	9	3	3	1	1	1	1
	Extensive	2	3	5	–	5	5	1	2	–	–	–	1
Size of lesion in mm	Average (min-max)	9 (2-20)	7 (2-20)	7 (1-30)	3 (2-10)	7 (2-25)	8 (1-50)	5 (2-10)	11 (2-20)	3 (1-4)	4 (2-10)	3 (2-4)	5 (2-10)
Lesion score	Sum	12	18	33	7	33	40	11	13	3	11	2	5

a PD, Parotid; SUB, Submandibular; RP, Retropharyngeal; PS, Prescapular; TRB, Tracheobronchial; MD, Mediastinal; PL, Portal; MS, Mesenteric; PF, Prefemoral; SUP, Supramammary or inguinal; MI, Medial Iliac; TR, Tracheal.

*Numbers in the table under each lymph node were frequency of encounter as per the type and scale of lesions, while lesion score was the sum of scores assigned for lesions observed for each lymph node*.

Eleven of the slaughtered cattle showed tuberculous lesions in their visceral organs: in the lungs (7/11), intestine (4/11), liver (3/11), thoracic membrane (2/11), abdominal membrane (1/11), kidney (1/11) and mammary gland (1/11). In the lungs, the most affected lobes were the cardiac lobe followed by the diaphragmatic lobe, while the accessory lobe was the least affected. One of these 11 animals was diagnosed with generalized tuberculosis and all four of its lung lobes were affected; two of the seven animals having lesions in their lungs were detected with lesion(s) in only the cardiac lobe, while lesion(s) in the remaining four animals were detected in the apical or diaphragmatic lobes.

Animal level lesion severity, as determined based on the sum of pathological scores, showed no clear and strong correlation with either BCS or the skin test thickness, whether considering the bovine (PPD-B) only reaction or the comparative (PPD-B minus PPD-A) test results (correlations not shown but all Meta data are listed in Supplementary Table 1).

### Species Identification

Mycobacterial culturing on LJ media yielded a total of 64 isolates, all confirmed deleted for RD4 (27), which is a characteristic feature for *M. bovis*. These isolates were obtained from 208 specimens collected from 27 slaughtered cattle with multiple isolates were recovered from several of the animals although no isolate was yielded from three of them. Of the 24 animals from which *M. bovis* was isolated, three animals were with non-visible lesions.

### Spoligotyping of *M. bovis* Strains

**Figure 3:**
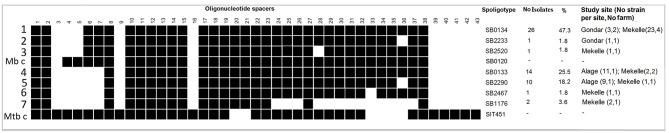
Isolates of *Mycobacterium bovis* (*n* = 55) obtained from tissues of SICCT reactor cattle grouped by spoligotyping. Spoligotype patterns are shown as 43 spacers in the DR region, either being present (black) or absent (white). Mb (*M. bovis*) - SB0120) and Mtb (*M. tuberculosis* strain H37Rv – SIT 451, http://www.pasteur-guadeloupe.fr:8081/SITVIT_ONLINE/query) were included as controls. Columns on the right side show the name of the spoligotype, its respective frequency of occurrence, and its corresponding proportion in study.

Spoligotype patterns were identified for 55 out of the 64 isolates and confirmed that all the isolates were *M. bovis*, while the results for the remaining were not interpretable due to either lower concentration or poor quality of genomic DNA in the samples. The identified spoligotypes lack spacers 3, 9, 16, and 39–43, a spoligotype signature unique to most *M. bovis* and is distinguished from other members of the *Mycobacterium tuberculosis* complex (MTC). The discriminatory power of the spoligotyping assay in the present study was moderately high (*HGDI* = 0.68). Considering identical spoligotypes from the same animal as one, a total of 28 unrelated strains were identified. The strains were grouped into seven spoligotypes, namely, SB0133, SB0134, SB1176, SB2233, SB2290, SB2467, and SB2520, defined after being compared with the global spoligotypes available in the *M. bovis* database (www.mbovis.org) (30). Spoligotype SB0134 was the most predominant spoligotype (47%, Figure 3). SB0133 was the second most frequently isolated spoligotype (25.5%), while SB2290 was the third frequently isolated spoligotype, accounting for 18% of the isolates.

Analysis of genetic similarity in the Direct Repeat (DR) region revealed that strains of SB0133, SB2290, SB2467, and SB1176 are likely related as marked by the lack of spacers 3–7, a characteristic of the Af2 clonal complex. On the other hand, strains of SB0134, SB2233, and SB2520 could not be categorized as Af2 strains as they had spacers 6 and 7 present (Figure 3).

Six of the identified spoligotypes, namely, SB0133, SB0134, SB1176, SB2290, SB2467, and SB2520, were from four herds that resided in Mekelle. Of these, one herd was recorded with only one spoligotype, two herds with two spoligotypes each, while the fourth herd had five spoligotypes recorded. In this fourth herd (herd size 100; 12% apparent prevalence), multiple strain types were identified in each of four of the 12 slaughtered reactor cattle, suggesting co-infection with at least two different strains of *M. bovis* in a single animal (Supplementary Table 1). This herd was established very recently and more than 90% of its cattle were sourced from known dairy herds or markets in Mekelle, Adigrat, and Humera. Trace backing the SICCT test history, some of the source herds had bTB infection history and can thereby be suspected as the main sources of infection for this herd from where we slaughtered the 12 reactors. In Gondar, spoligotypes SB0134 and SB2233 were identified from two herds, one from each, while in Alage spoligotypes SB2290 and SB0133 were identified from that single herd. The geographical locations of the dairy herds, where specific spoligotypes were found, are embedded in Figure 1.

## Discussion

A recent review on the extent of bTB in Ethiopia demonstrated that bTB has become a major concern in dairy cattle managed under intensive and semi-intensive production systems, severely constraining this sector (8). Sound bTB control policy necessitates the availability of scientific knowledge on the epidemiology of the disease. The major risk factors for promoting bTB transmission across the country are related to cattle movement (19); however, to date Ethiopia has no policy on animal movement regulation and traceability systems. This has posed the need for scrutinizing the genotypic and geographic distribution of *M. bovis* strains that cause disease in cattle. In this study, we attempted to explore the extent of tuberculous lesions in dairy cattle in selected sites, isolate, and characterize the *M. bovis* strains causing the disease, and describe their role in the epidemiology of bTB.

It is understandable that periodic, pathology-based re-evaluations of diseases facilitate the identification of any alterations in their character or severity (32, 33). One relevant finding in this regard is the lesion distribution, which we believe add values on situations in Ethiopian dairy cattle infected under natural conditions and with no eradication programme being in place. Eradication campaigns in several countries decreased the number of bTB cases showing extensive lesions and more frequent lesion encounter in thoracic lymph nodes than lung parenchyma (33). In this study, tuberculous lesions were more frequently encountered in the thoracic region compared to other body parts, suggesting the respiratory route of *M. bovis* transmission (34). More severe lesions were found to occur in higher frequency in the lungs and lymph nodes along the respiratory track, which were consistent with the data produced in the UK, USA, and Australia before implementation of bTB control programme (33).

The SICCT test results and post-mortem investigations of the presented study showed that not all SICCT test reactor animals had developed lesions, i.e., 21 out of 27 slaughtered cattle showed typical tuberculous lesions, while the remaining six did not. The lack of pathology in some reactor cattle could be a reflection of the short period between time of infection and the test and post-mortem events (35–38).

Positive correlations between SICCT test status and post-mortem tuberculous lesions has been reported previously (37, 39, 40); however, there are limited studies to show a correlation between skin thickness measurements from SICCT reactors and severity of pathology. One study in Ethiopia (9) provided a weak negative correlation, while our study demonstrated no clear and strong correlation between lesion severity and the skin test thickness, whether considering the bovine only reaction, or the SICCT result. The small sample size and non-randomized selection of sacrificed animals could have introduced some level of bias in the current study. Several other factors such as variation in the level of prevalence, breed of cattle involved, and the age distribution may also affect this trend. For example, it is not uncommon that chronically infected animals with severe pathology are unresponsive for the SICCT test (20, 41). On the other hand, animals at early stage of infection tend to show optimal skin reaction but with fewer and less severe pathology (Supplementary Table 1). These suggest that the skin reaction for the SICCT test does not necessarily tell the extent of pathology within the animals (42); however, it requires further evidence to substantiate this.

Finding in this study creates a nice link of data between slaughter and mycobacterial identification. The *Mycobacterium* species isolated from tuberculous lesions in this study were only *M. bovis* with a culturing yield of 89% (24/27), which is comparable with previous reports (9, 33). Isolation of *M. bovis* from three cattle with non-visible lesions in this study shows that there are limitations in proving the infection status of SICCT positive animals despite thorough post-mortem inspection. Therefore, to understand the true bTB status of an animal, culturing lymph nodes (particularly retropharyngeal, bronchial, and mediastinal lymph nodes) from any SICCT reactor animals are of value, even in the absence of visible lesions. In contrast to other studies in Ethiopia (10, 27, 43), neither *M. tuberculosis* strains nor other *Mycobacterium* species such as non-tuberculous mycobacteria were isolated in the present study. This could be related to a low extent of other MTC and environmental mycobacteria in the study areas, or the number of slaughtered cattle might not have been sufficient enough to detect such mycobacterial species. It is known that other *Mycobacterium* species of the MTC, such as *M. tuberculosis, M. caprae*, and *M. orygis*, can cause infection and be isolated from SICCT test reactor cattle as this test is not specific to *M. bovis* (9, 10, 43–48).

The 55 *M. bovis* strains that were spoligotyped as well as confirmed to be deleted for RD4 could be stratified into 7 spoligotype patterns. Of these, SB0133 and SB0134 were the most predominant types in the study areas. These findings are in line with reports for central Ethiopia where SB0133, SB0134, and SB1176 have been reported in high frequencies by several authors (9, 10, 27, 49, 50). Historical data on dairy cattle trade that goes back to late 1990ies (19) suggest that cattle have, to significant extent, been traded from central Ethiopia, where the bTB burden has been high, to Mekelle and remaining parts of Tigray regional state, and thus, identifying strains with the same spoligotypes in our study areas support epidemiological links and suggest that cattle movement could have had a significant role in such transmission. Among the other spoligotypes, patterns named as SB2233, SB2290, SB2467, and SB2520 have not been reported before in Ethiopia. However, these spoligotypes are likely derivatives from the dominating types (as they lack only one additional spacer), strains of these types might have evolved more recently and could therefore be less prevalent and distributed.

The diversity of strains found in the present study substantiates that spoligotyping is a valuable tool for bTB epidemiological studies, even if a discriminatory power (HGDI) of 0.68 in this study is lower than previous reports (51, 52). The diversity could be a reflection of the moderately high bTB prevalence in the study areas that leads to constricted transmission of most strains except for SB0134, which was isolated in larger proportions of strains from more than 70% of the investigated herds compared to other herds (Supplementary Table 1). SB0133, though accounting for a larger proportion of the strains (47%), was restricted to three of the seven herds, unlike SB0134, which was identified in five of them. This variation in distribution of spoligotypes across regions and herds could be linked to either or both of the following points: (1) sampling bias as animal recruitment for slaughter was based on animal owners' willingness; (2) difference in inherent ability (genetic advantage) between strains on transmissibility (52–54). Linked to the latter, strains with SB0134 were identified in almost all herds where *M. bovis* was isolated in Gondar and Mekelle and that could support a high transmissibility for this strain type; future research may clarify any such difference among *M. bovis* types.

Strains with spoligotype SB0134 are found worldwide (55, 56). It has been found also in Eritrea (57), Mali (58), South Africa (59), and several European countries including UK (60), France (61), and Spain (62). It seems probable that strains clustered as SB0134 in Ethiopia could share a common ancestor with strains from continental Europe and that introduction into Ethiopia could have occurred along with imported dairy cows from Europe and time since the advent of dairy development in Ethiopia around 1947 (51, 63). However, the phylogenetic relationship between strains of SB0134 in Africa and Europe has not yet been demonstrated; whole genome sequencing of these strains may give clue to their relationship. The history of SB0133 is also difficult to interpret. However, as SB0133 is so far only common in Ethiopia and some other African countries, such as Uganda and Tanzania (55), it may have evolved in Africa and not spread beyond this region. SB1176 was first reported in Ethiopia (47) and there is no report indicating its existence outside of Ethiopia, not even in Eritrea (57).

All *M. bovis* strains lack at least spacers 3, 9, 16, and 39–41 (64). Many strains of this study clustered into four spoligotypes that also lack spacers 4–7 (SB0133, SB1176, SB2290, and SB2467), deletions that mark the *M. bovis* Af2 clonal complex, which is present only in East Africa (55) and thus it is not surprising to find these types represented. Other spoligotypes represented in this study were SB0134, SB2233, and SB2520, did not have spoligotype patterns that mark Af2 or any of the three other defined *M. bovis* clonal complexes, namely, Eu1, Eu2, and Af1 (56, 65, 66), suggesting that the strains with such spoligotype patterns may have a different evolutionary history (9), and could reflect the limitations of the currently defined clonal complexes to represent the entire diversity of *M. bovis*. Such limitations, however, may be resolved in this era of genomics where whole genome sequencing technology can be utilized to define new clonal complexes or lineages. With respect to the evolutionary scenario for this group of spoligotypes, the strains defined in this so called “Other” clonal complex might have descended from SB0134, as the other two spoligotypes share similar spoligotype patterns. However, how the current population structure of *M. bovis* has evolved in the East African region is difficult to trace. One possibility could be by drift during the spread of strains between regions in a series of founder events, or subsequently as the population expanded and related to cattle movement within and/or between the region (19, 55, 56).

In conclusion, despite a small sample size, the present study showed a strain diversity that could be classified into seven spoligotypes with multiple genotypes identified in a single herd or even in a single animal and with no sign of geographical localization of these genotypes. This could be a consequence of frequent and significant movement of bTB diseased cattle around the country, spreading the disease. Any future control of bTB in Ethiopia needs to address the risks of cattle movement.

## Data Availability Statement

All data generated and/or analyzed during the current study are not publicly available but are available from the corresponding author on reasonable request.

## Ethics Statement

The research approval was granted by the Institutional Review Board (IRB) of Aklilu Lemma Institute of Pathobiology, Addis Ababa University (Reference number IRB/ALIPB/2018). Implementation of the study was supported by the Ethiopian Ministry of Agriculture. SICCT testing of cattle was done based on the OIE standards for skin testing (20), and performed after a verbal informed consent was obtained from cattle owners as most animal keepers were illiterate, and so standard written informed consent approaches were not relevant. Besides, bTB reactor animals identified for post-mortem inspections were transported and slaughtered based on a standard guideline for animal handling, transport, and slaughter (67).

## Author Contributions

GM performed the research, analyzed the data, and wrote the paper. SB, AM, BG, GA, and JW contributed to the study conception and design and reviewed the paper. ML, MT, AO, AA, MS, and EH performed field and laboratory tests. All authors contributed to the article and approved the submitted version.

## Conflict of Interest

The authors declare that the research was conducted in the absence of any commercial or financial relationships that could be construed as a potential conflict of interest.

## Abbreviations

Af1: African 1
Af2: African 2
ATVET: Agricultural Technical and Vocational Education Training College
bTB: Bovine tuberculosis
DR: Direct repeat
ETHICOBOTS: Ethiopia Control of Bovine Tuberculosis Strategies
Eu1: European 1 Clonal Complex
Eu2: European 2 Clonal Complex
HGDI: Hunter Gaston Discrimination Index
MTC: *Mycobacterium tuberculosis* complex
SICCT: Single Intradermal Comparative Cervical Tuberculin
RD: Region of Difference.

## References

[1] Cousins DV. *Mycobacterium bovis* infection and control in domestic livestock. Rev Sci Tech Off Int Epiz. (2001) 20:71–85. 10.20506/rst.20.1.1263 11288521

[2] More S Bøtner A Butterworth A Calistri P Depner K Edwards S. Assessment of listing and categorization of animal diseases within the framework of the animal health law (Regulation (EU) No 2016/429): bovine tuberculosis. EFSA. (2017) 15:04959. 10.2903/j.efsa.2017.4959 32625624 PMC7009898

[3] Kaneene JB Pfeiffer D Epidemiology of *Mycobacterium bovis*. In: Thoen CO, Steele JH, Gilsdorf MJ, editors. Mycobacterium Bovis Infection in Animals and Humans. Blackwell Publishing Ltd. (2006). p. 34–48. 10.1002/9780470344538.ch5

[4] Allen AR Skuce RA Byrne AW Bovine tuberculosis in Britain and Ireland - a perfect storm? The confluence of potential ecological and epidemiological impediments to controlling a chronic infectious disease. Front Vet Sci. (2018) 5:109 10.3389/fvets.2018.00109 29951489 PMC6008655

[5] Humblet MF Boschiroli ML Saegerman C. Classification of worldwide bovine tuberculosis risk factors in cattle: a stratified approach. Vet Res. (2009) 40:50. 10.1051/vetres/2009033 19497258 PMC2710499

[6] Zinsstag J Schelling E Roth F Kazwala RR Economics of bovine tuberculosis. In: Thoen CO, Steele JH, Gilsdorf MJ, editors. Mycobacterium Bovis Infection in Animals and Humans. 2nd ed. Ames, IA: Iowa State University Press (2006). p. 68–83. 10.1002/9780470344538.ch9

[7] Teppawar RN Chaudhari S Moon SL Shinde S Khan WA Patil AR Zoonotic Tuberculosis: A Concern and Strategies to Combat 2018. Available online at: https://www.intechopen.com/books/basic-biology-and-applications-of-actinobacteria/zoonotic-tuberculosis-a-concern-and-strategies-to-combat (accessed February 11, 2020). 10.5772/intechopen.76802

[8] Sibhat B Asmare K Demissie K Ayelet G Mamo G Ameni G. Bovine tuberculosis in Ethiopia: a systematic review and meta-analysis. Prev Vet Med. (2017) 147:149–57. 10.1016/j.prevetmed.2017.09.006 29254713 PMC5739073

[9] Firdessa R Tschopp R Wubete A Sombo M Hailu E Erenso G. High prevalence of bovine tuberculosis in dairy cattle in central Ethiopia: implications for the dairy industry and public health. PLoS ONE. (2012) 7:e52851. 10.1371/journal.pone.0052851 23285202 PMC3532161

[10] Tsegaye W Aseffa A Mache A Mengistu Y Berg S Ameni G Conventional and molecular epidemiology of bovine tuberculosis in dairy farms in addis Ababa city, the capital of ethiopia. Intern J Appl Res Vet Med. (2010) 8:143–51.

[11] Elias K Hussein D Gebeyehu M. Status of bovine tuberculosis in addis Ababa dairy farms. Rev Sci Tech Off Int Epiz. (2008) 27:915–23. 10.20506/rst.27.3.1850 19284060

[12] Ameni G Aseffa A Engers H Young D Gordon S Hewinson G. High prevalence and increased severity of pathology of bovine tuberculosis in Holsteins compared to zebu breeds under field cattle husbandry in central Ethiopia. Clin Vaccine Immunol. (2007) 14:1356–61. 10.1128/CVI.00205-07 17761523 PMC2168113

[13] Ameni G Bonnet P Tibbo M A cross-sectional study of bovine tuberculosis in selected dairy farms in ethiopia. Int J App Res Vet Med. (2003) 1:253–8.

[14] Mekonnen GA Conlan AJK Berg S Ayele BT Alemu A Guta S. Prevalence of bovine tuberculosis and its associated risk factors in the emerging dairy belts of regional cities in Ethiopia. Prev Vet Med. (2019) 168:81–9. 10.1016/j.prevetmed.2019.04.010 31097127 PMC10364076

[15] Hadush GA Assessment of Bovine Tuberculosis in Dairy Farms and its Public Health Importance in and Around Adigrat District, in Food Safety and Zoonosis. [Master's thesis]. Mekelle University, Mekelle, Ethiopia.

[16] Wubete A Almaw G Koran T Tamiru M Sahle M Mekonnen GA Bovine Tuberculosis Study in Farmed Dairy Cattle and Cattle Genetic, Improvement Centers in Selected Areas of Ethiopia. Poster Abstracts - Epidemiology - British Cattle Veterinary Association. (2015) Available online at: https://www.bcva.eu/system/files/resources/Epidemiology (accessed May 20, 2019).

[17] Romha G Gobena A Berhe G Mamo G Epidemiology of mycobacterial infections in cattle in two districts of Western tigray zone, Northern Ethiopia. Afr J Microbiol Res. (2013) 7:4031–8. 10.5897/AJMR12.1951

[18] Regassa A Tassew A Amenu K Megersa B Abunna F Mekibib B. A cross-sectional study on bovine tuberculosis in Hawassa town and its surroundings, Southern Ethiopia. Trop Anim Health Prod. (2010) 42:915–20. 10.1007/s11250-009-9507-4 19957029

[19] Mekonnen GA Ameni G Wood JLN The ETHICOBOTS consortium Berg S Conlan AC. Network analysis of dairy cattle movement and its implication onassociations with Bovine Tuberculosis spread and control in emerging dairy belts of Ethiopia. BMC Vet Res. (2019) 15:262. 10.1186/s12917-019-1962-1 31349832 PMC6660945

[20] OIE Bovine tuberculosis. Manual of Diagnostic Tests and Vaccines for Terrestrial Animals. International Organization for Animal Health (OIE). (2009). Available online at: www.oie.int (accessed September 30, 2019)

[21] Kellogg W Body Condition Scoring With Dairy Cattle- FSA4008. Agriculture and Natural Resources, University of Arkansas Cooperative Extension Service (2010) Available online at: https://www.uaex.edu/publications/pdf/FSA-4008.pdf (accessed January 28, 2016).

[22] Ameni G Aseffa A Engers H Young D Hewinson G Vordermeier M. Cattle husbandry in Ethiopia is a predominant factor affecting the pathology of bovine tuberculosis and gamma interferon responses to mycobacterial antigens. Clin Vaccine Immunol. (2006) 13:1030–6. 10.1128/CVI.00134-06 16960115 PMC1563579

[23] Vordermeier HM Chambers MA Cockle PJ Whelan AO Simmons J Hewinson RG. Correlation of ESAT-6-specific gamma interferon with pathology in cattle following *Mycobacterium bovis* BCG vaccination against experimental bovine tuberculosis. Infect Immun. (2002) 70:3026–32. 10.1128/IAI.70.6.3026-3032.2002 12010994 PMC128013

[24] Roberts GD Koneman EW Kim YK Mycobacterium. In: Balow A, editor. Manual of Clinical Microbiology. Washington, DC: American Society for Microbiology (1991). p. 304–39.

[25] Qiagen QIAamp DNA Mini and Blood Mini Handbook. 5th ed. Sample and Assay Technologies (2016). Available online at: http://www.qiagen.com (accessed March 30, 2019).

[26] Brosch R Gordon SV Marmiesse M Brodin P Buchrieser C Eiglmeier K. A new evolutionary scenario for the *Mycobacterium tuberculosis* complex. PNAS. (2002) 99:3684–9. 10.1073/pnas.052548299 11891304 PMC122584

[27] Berg S Firdessa R Habtamu M Gadisa E Mengistu A Yamuah L. The burden of mycobacterial disease in Ethiopian cattle: implications for public health. PLoS ONE. (2009) 4:e5068. 10.1371/journal.pone.0005068 19352493 PMC2662418

[28] Kamerbeek J Schouls L Kolk A van Agterveld M van Soolingen D Kuijper S. Simultaneous detection and strain differentiation of *Mycobacterium tuberculosis* for diagnosis and epidemiology. J Clin Microbiol. (1997) 35:907–14. 10.1128/JCM.35.4.907-914.1997 9157152 PMC229700

[29] Molhuizen HOF Bunschoten AE Schouls LM van Embden JDA. Rapid Detection and Simultaneous Strain Differentiation of *Mycobacterium tuberculosis* Complex Bacteria by Spoligotyping. In: Parish T, Stoker NG, editors. Mycobacteria Protocols. Methods in Molecular Biology™. Vol. 101. Humana Press (1998). p. 381–94. 10.1385/0-89603-471-2:381 9921492

[30] Smith NH Upton P. Naming spoligotype patterns for the RD9-deleted lineage of the *Mycobacterium tuberculosis* complex; www.Mbovis.org. Infect Genet Evol. (2012) 12:873–6. 10.1016/j.meegid.2011.08.002 21855653

[31] Hunter PR Gaston MA. Numerical index of the discriminatory ability of typing systems: an application of Simpson's index of diversity. J Clin Microbiol. (1988) 26:2465–6. 10.1128/JCM.26.11.2465-2466.1988 3069867 PMC266921

[32] Cassidy JP. The pathology of bovine tuberculosis: time for an audit. Vet J. (2008) 176:263–4. 10.1016/j.tvjl.2007.09.001 17936047

[33] Liebana E Johnson L Gough J Durr P Jahans K Clifton-Hadley R. Pathology of naturally occurring bovine tuberculosis in England and Wales. Vet J. (2008) 176:354–60. 10.1016/j.tvjl.2007.07.001 17728162

[34] Thoen CO Bloom BR Pathogenesis of *Mycobacterium bovis*. In: Thoen CO, Steele JH, editors. Mycobacterium Bovis Infection in Animals and Humans. Ames, IA: Iowa State University Press (1995). p. 3–14.

[35] Phillips CJ Foster CR Morris PA Teverson R. The transmission of *Mycobacterium bovis* infection to cattle. Res Vet Sci. (2003) 74:1–15. 10.1016/S0034-5288(02)00145-5 12507561

[36] Abdelaal HFM Spalink D Amer A Steinberg H Hashish EA Nasr EA. Genomic polymorphism associated with the emergence of virulent isolates of *Mycobacterium bovis* in the Nile Delta. Sci Rep. (2019) 9:11657. 10.1038/s41598-019-48106-3 31406159 PMC6690966

[37] O'Hagan MJ Courcier EA Drewe JA Gordon AW McNair JA bernethy DA Risk factors for visible lesions or positive laboratory tests in bovine tuberculosis reactor cattle in Northern Ireland. Prev Vet Med. (2015) 120:283–90. 10.1016/j.prevetmed.2015.04.005 25957973

[38] Whipple DL Bolin CA Miller JM. Distribution of lesions in cattle infected with *Mycobacterium bovis*. J Vet Diagn Invest. (1996) 8:351–4. 10.1177/104063879600800312 8844579

[39] Byrne AW Graham J Brown C Donaghy A Guelbenzu-Gonzalo M McNair J. Bovine tuberculosis visible lesions in cattle culled during herd breakdowns: the effects of individual characteristics, trade movement and co-infection. BMC Vet Res. (2017) 13:400. 10.1186/s12917-017-1321-z 29284483 PMC5747088

[40] Clegg TA Good M More SJ. Risk factors for cattle presenting with a confirmed bTB lesion at slaughter, from herds with no evidence of within-herd transmission. Prev Vet Med. (2016) 126:111–20. 10.1016/j.prevetmed.2016.02.003 26895647

[41] Houlihan M Dixon FW Page NA. Outbreak of bovine tuberculosis featuring anergy to the skin test, udder lesions and milk borne disease in young calves. Vet Rec. (2008) 163:357–61. 10.1136/vr.163.12.357 18806280

[42] de la Rua-Domenech R Goodchild AT Vordermeier HM Hewinson RG Christiansen KH Clifton-Hadley RS. Ante mortem diagnosis of tuberculosis in cattle: a review of the tuberculin tests, gamma-interferon assay and other ancillary diagnostic techniques. Res Vet Sci. (2006) 81:190–210. 10.1016/j.rvsc.2005.11.005 16513150

[43] Ameni G Vordermeier M Firdessa R Aseffa A Hewinson G Gordon SV. *Mycobacterium tuberculosis* infection in grazing cattle in central Ethiopia. Vet J. (2011) 188:359–61. 10.1016/j.tvjl.2010.05.005 20965132 PMC3103825

[44] Kuria JKN Diseases Caused by Bacteria in Cattle: Tuberculosis. IntechOpen (2019).

[45] Dawson KL Bell A Kawakami RP Coley K Yates G Collins DM. Transmission of *Mycobacterium orygis* (*M. tuberculosis* complex species) from a tuberculosis patient to a dairy cow in New Zealand. J Clin Microbiol. (2012) 50:3136–8. 10.1128/JCM.01652-12 22785186 PMC3421823

[46] Gathogo SM Kuria JKN Ombui JN. Prevalence of bovine tuberculosis in slaughter cattle in Kenya: a postmortem, microbiological and DNA molecular study. Trop Anim Health Pro. (2012) 44:1739–44. 10.1007/s11250-012-0131-3 22528528

[47] Ameni G Desta F Firdessa R. Molecular typing of *Mycobacterium bovis* isolated from tuberculosis lesions of cattle in north eastern Ethiopia. Vet Rec. (2010) 167:138–41. 10.1136/vr.b4881 20656993

[48] de Jong BC Antonio M Gagneux S. *Mycobacterium africanum -* review of an important cause of human tuberculosis in West Africa. PLoS Negl Trop Dis. (2010) 4:e744. 10.1371/journal.pntd.0000744 20927191 PMC2946903

[49] Ameni G Bekele S Tolosa T Preliminary study on the impact of Bovine tuberculosis on the reproductive efficiency and productivity of Holstein dairy cows in Central Ethiopia. Bull Anim Hlth Prod Afr. (2010) 58:223–8. 10.4314/bahpa.v58i3.64210

[50] Romha G Gebru G Asefa A Mamo G. Epidemiology of *Mycobacterium bovis* and *Mycobacterium tuberculosis* in animals: transmission dynamics and control challenges of zoonotic TB in Ethiopia. Prev Vet Med. (2018) 158:1–17. 10.1016/j.prevetmed.2018.06.012 30220382

[51] Biffa D Skjerve E Oloya J Bogale A Abebe F Dahle U. Molecular characterization of *Mycobacterium bovis* isolates from Ethiopian cattle. BMC Vet Res. (2010) 27:6–28. 10.1186/1746-6148-6-28 20507576 PMC2886024

[52] Duarte EL Domingos M Amado A Botelho A. Spoligotype diversity of *Mycobacterium bovis* and *Mycobacterium caprae* animal isolates. Vet Microbiol. (2008) 130:415–21. 10.1016/j.vetmic.2008.02.012 18417301

[53] Skuce RA Neill SD. Molecular epidemiology of *Mycobacterium bovis*: exploiting molecular data. Tuberculosis. (2001) 81:169–75. 10.1054/tube.2000.0270 11463239

[54] van Embden JDA van Gorkom T Kremer K Jansen R van der Zeijst BAM Schouls LM. Genetic variation and evolutionary origin of the direct repeat locus of *Mycobacterium tuberculosis* complex bacteria. J Bacteriol. (2000) 182:2393–401. 10.1128/JB.182.9.2393-2401.2000 10762237 PMC111299

[55] Berg S Garcia-Pelayo MC Muller B Hailu E Asiimwe B Kremer K. African 2, a clonal complex of *Mycobacterium bovis* epidemiologically important in East Africa. J Bacteriol. (2011) 193:670–8. 10.1128/JB.00750-10 21097608 PMC3021238

[56] Müller B Hilty M Berg S Garcia-Pelayo MC Dale J Boschiroli ML. African 1, an epidemiologically important clonal complex of *Mycobacterium bovis* dominant in Mali, Nigeria, Cameroon, and Chad. J Bacteriol. (2009) 191:1951–60. 10.1128/JB.01590-08 19136597 PMC2648362

[57] Ghebremariam MK Hlokwe T Rutten VPMG Allepuz A Cadmus S Muwonge A. Genetic profiling of *Mycobacterium bovis* strains from slaughtered cattle in Eritrea. PLoS Negl Trop Dis. (2018) 12:e0006406. 10.1371/journal.pntd.0006406 29664901 PMC5922621

[58] Müller B Steiner B Bonfoh B Fane A Smith NH Zinsstag J. Molecular characterisation of *Mycobacterium bovis* isolated from cattle slaughtered at the Bamako abattoir in Mali. BMC Vet Res. (2008) 4:26. 10.1186/1746-6148-4-26 18637160 PMC2483712

[59] Michel AL Hlokwe TM Coetzee ML Mare L Connoway L Rutten VP. High *Mycobacterium bovis* genetic diversity in a low prevalence setting. Vet Microbiol. (2008) 126:151–9. 10.1016/j.vetmic.2007.07.015 17720336

[60] Gibson AL Hewinson G Goodchild T Watt B Story A Inwald J. Molecular epidemiology of disease due to *Mycobacterium bovis* in humans in the United Kingdom. J Clin Microbiol. (2004) 42:431–4. 10.1128/JCM.42.1.431-434.2004 14715798 PMC321667

[61] Haddad N Ostyn A Karoui C Masselot M Thorel MF Hughes SL. Spoligotype diversity of *Mycobacterium bovis* strains isolated in France from 1979 to 2000. J Clin Microbiol. (2001) 39:3623–32. 10.1128/JCM.39.10.3623-3632.2001 11574583 PMC88399

[62] Aranaz A Liebana E Mateos A Dominguez L Vidal D Domingo M. Spacer oligonucleotide typing of *Mycobacterium bovis* strains from cattle and other animals: a tool for studying epidemiology of tuberculosis. J Clin Microbiol. (1996) 34:2734–40. 10.1128/JCM.34.11.2734-2740.1996 8897175 PMC229396

[63] Felleke G Milk and Dairy Products, Post-Harvest Losses and Food Safety in Sub-Saharan Africa and the Near East. A Review of the Small Scale Dairy Sector – Ethiopia. FAO Prevention of Food Losses Programme. Rome: FAO (2003).

[64] Smith NH Gordon SV de la Rua-Domenech R Clifton-Hadley RS Hewinson RG. Bottlenecks and broomsticks: the molecular evolution of *Mycobacterium bovis*. Nat Rev Microbiol. (2006) 4:670–81. 10.1038/nrmicro1472 16912712

[65] Rodriguez-Campos S Schürch AC Dale J Lohan AJ Cunha MV Botelho A. European 2–a clonal complex of *Mycobacterium Bovis* dominant in the Iberian Peninsula. Infect Genet Evol. (2012) 12:866–72. 10.1016/j.meegid.2011.09.004 21945286

[66] Smith NH Berg S Dale J Allen A Rodriguez S Romero B. European 1: a globally important clonal complex of *Mycobacterium bovis*. Infect Genet Evol. (2011) 11:1340–51. 10.1016/j.meegid.2011.04.027 21571099

[67] Chambers PG Grandin T Guidelines for Humane Handling, Transport and Slaughter of Livestock. Bangkok: Food and Agriculture Organization of the United Nations, Regional Office for Asia and the Pacific (2001). p. 49–68.

