# Supplementary material for: Genotype Diversity of Mycobacterium bovis and Pathology of Bovine Tuberculosis in Selected Emerging Dairy Regions of Ethiopia
Source: Front Vet Sci. 2020 Sep 30;7:553940. doi: 10.3389/fvets.2020.553940 (PMC7554335; doi:10.3389/fvets.2020.553940)
Supplement: Supplementary file 1 [file Table_1.docx]

**Supplementary Table S1:** Meta data of 27 animals and *Mycobacterium bovis* isolates from respective animal.

| **Farm ID (within-herd prevalence)** | **Animal ID** | **Age (year)** | **Breed** | **Sex** | **BCS** | **Skin thickness (mm) ^d^** | **Sample source** | **Lesion score (SUM) ^e^** | **SB No** | **Animal origin and BTB status of the source** |
| --- | --- | --- | --- | --- | --- | --- | --- | --- | --- | --- |
| 8F026 (12%) | 8A0431 ^a^ | 5 | HC | F | 3 | 4.44 | MD, TRB | 9 | SB2467, B0133 | Farm ID, 8F016, Mekelle city, within-herd prevalence of the source herd was 82%. ^b^ |
|  | 8A0407 | 3 | HC | F | 2 | 9.95 | TRB | 5 | SB0134 |  |
|  | 8A0409 | 3 | HC | F | 2 | 15.95 | MD | 5 | SB0134 |  |
|  | 8A0438 ^a^ | 6 | HC | F | 2 | 8.25 | MD, TRB | 4 | SB0133, B2290 | Farm ID, 8F007, Mekelle city, herd prevalence in the 2018 SICCT test was 2.5%, the farm had practice of removing less productive and older cattle. ^b^ |
|  | 8A0429 | 3 | HC | F | 2 | 4.43 | MD, TRB | 9 | SB0133 | These animals were originated from Adigrat where the prevalence was 11.7% (15). |
|  | 8A0459 | 6 | HC | F | 2 | 6.01 | MD | 7 | SB0133 |  |
|  | 8A0415 | 1.5 | HC | F | 2 | 11.77 | Lung | 12 | SB0134 | Farm ID, 8F019, Mekelle city, within-herd prevalence of the source herd was 5.9%. ^b^ |
|  | 8A0449 ^a^ | 0.3 | HC | F | 2 | 4.14 | MD, TRB | 9 | SB0133, B2520 | These animals were born in the same farm; within-herd prevalence was 12%. ^b^ |
|  | 8A0448 | 1 | HC | F | 2 | 13.12 | MD, TRB | 14 | SB0134 |  |
|  | 8A0597 ^a^ | 0.08 | HC | M | 2 | 8.05 | Lung | 2 | SB0133, B0134 |  |
|  | 8A0404 | 2.5 | HC | F | 2 | 16.21 | Lung, MS | 0 | - |  |
|  | 8A0453 | 2.5 | HC | F | 2 | 15.62 | Lung, RP | 0 | - |  |
| 8F018 (94%) | 8A0315 | 3.5 | HC | F | 2 | 13.32 | MD, TRB | 10 | SB0134 | Bought from other places with no information on BTB status of the source herd. ^b^ |
|  | 8A0320 | 5 | HC | F | 1 | 7.27 | PD | 69 | SB0134 | Born in the same farm, within-herd prevalence was 93.8%. ^b^ |
| 8F002 (77%) | 8A0036 | 0.7 | HC | M | 2 | 7.63 | MD | 17 | SB1176 | These animals were born on the same farm, within herd prevalence was 15.6% (16); later increased to 77%. |
|  | 8A0035 | 1 | HC | F | 2 | 7.57 | MD | 12 | SB0134 |  |
| 8F038 (4.2%) | 8A0574 | 5.7 | HC | F | 2 | 14.05 | MD, TRB | 8 | SB0134 | Born in the same farm, within-herd prevalence was 4.2%. ^b^ |
| 7F053 (11%) | 7A0855 | 1.33 | HC | F | 2 | 9.15 | Lung | 11 | SB2233 | Born in the same farm, herd frequently graze on field with possibility of mingling with other herds, within-herd prevalence was 11%. ^b^ |
| 7F013 (1.7%) | 7A0168 | 2 | HC | F | 1 | 7.14 | Lung, MD | 2 | SB0134 | Born in the same herd, its dam dead of coughing, within-herd prevalence was 10.6% (16). |
| 7F039 (5%) ^c^ | 7A0591 | 1.5 | HC | F | 3 | 6.87 | MD | 0 | - | ^b^ Born in the same herd, its dam was a reactor for SICCT, within-herd prevalence was 5%. The isolate deleted RD4 but not confirmed by spoligotyping. |
| 7F018 (13.3%) ^c^ | 7A0226 | 4 | HC | F | 1 | 6.23 | Lung, MD | 0 | - | Born in the same herd, within-herd prevalence was 13.3%. ^b^  The isolate deleted RD4 but not confirmed by spoligotyping. |
| 7F027 (1.2%)^c^ | 7A0366 | 9.25 | HC | F | 2 | 5.66 | Lung, MD | 0 | - | Born in the same herd, within-herd prevalence was 1.2%. ^b^  The isolate deleted RD4 but not confirmed by spoligotyping. |

| 10F01 (4.6%) | AB720 ^a^ | 3 | Z | F | 3 | 8.92 | MD, TRB | 3 | SB2290, SB0133 | These animals were born in the same herd, its dam brought from Borana (Southern Ethiopia); within-herd prevalence was 5.2%. ^b^ |
| --- | --- | --- | --- | --- | --- | --- | --- | --- | --- | --- |
|  | AB730 | 2.75 | Z | F | 3 | 7.4 | RP | 6 | SB2290 |  |
|  | AB394 | 9.17 | Z | F | 2 | 5.11 | RP | 10 | SB2290 |  |
|  | AB312 | 11.83 | Z | F | 2 | 7.99 | MD, TRB | 17 | SB2290 |  |
|  | AB737 |  | Z | F | 3 | 9.6 | MD, TRB | 0 | - |  |

*Note:* Farm IDs starting with number 8, 7, and 10 were from Mekele, Gondar and Alage, respectively; Correlation between BCS and lesion severity is weakly inversely related (*r* = -0.17).

*Abbreviations*: AAC - Alage Agricultural College; BCS - body condition scoring; HC - Holstein Frisian and Zebu cross breed; Z - Zebu breed; PD- Parotid; RP-Retropharyngeal; TRB-Tracheobronchial; MD-Mediastinal, MS- Mesenteric lymph nodes

^a^ animals co-infected with two strains of *M. bovis;*

^b^ Farm specific BTB prevalence data were summarized in Mekonnen *et al.* (14);

^c^ Isolate from these animals were confirmed to deleted RD4 but the spoligotype patterns were not interpretable.

^d^ skin thickness based on standard interpretation of the SICCT test rated as described in Mekonnen *et al.* (14);

^e^ sum of all pathological scores (visceral organs and lymph nodes)
